# Recomendações para o Manejo de Dispositivos Cardíacos Eletrônicos Implantáveis *Post Mortem*

**DOI:** 10.36660/abc.20200447

**Published:** 2020-12-01

**Authors:** Júlio César de Oliveira, Alexsandro Alves Fagundes, Ricardo Alkmim-Teixeira, José Mário Baggio, Luciana Armaganijan, Andre d’Avila, Eduardo B. Saad, Veridiana Silva de Andrade, Luis Gustavo Belo de Moraes, Ricardo Kuniyoshi, André Gustavo da Silva Rezende, Mauricio Pimentel, Thiago da Rocha Rodrigues, Helio Lima de Brito, Elenir Nadalin, Cristiano Faria Pisani, Elerson Arfelli, Fatima Dumas Cintra, Carlos Antonio Abunader Kalil, Sissy Lara de Melo, Priscila Moreno Sperling Cannavan

**Affiliations:** 1 Universidade Federal de Mato Grosso CuiabáMS Brasil Universidade Federal de Mato Grosso, Cuiabá, MS - Brasil; 2 Hospital Geral Filantrópico Universitário de Cuiabá CuiabáMS Brasil Hospital Geral Filantrópico Universitário de Cuiabá, Cuiabá, MS - Brasil; 3 Universidade do Estado da Bahia SalvadorBA Brasil Universidade do Estado da Bahia, Salvador, BA - Brasil; 4 Hospital Renascentista Pouso AlegreMG Brasil Hospital Renascentista, Pouso Alegre, MG - Brasil; 5 Universidade do Vale do Sapucaí Pouso AlegreMG Brasil Universidade do Vale do Sapucaí, Pouso Alegre, MG - Brasil; 6 Instituto de Cardiologia do Distrito Federal BrasíliaDF Brasil Instituto de Cardiologia do Distrito Federal,Brasília, DF - Brasil; 7 Instituto Dante Pazzanese de Cardiologia São PauloSP Brasil Instituto Dante Pazzanese de Cardiologia,São Paulo, SP - Brasil; 8 Hospital SOS Cardio FlorianópolisSC Brasil Hospital SOS Cardio, Florianópolis, SC - Brasil; 9 Hospital Pró-Cardíaco Rio de JaneiroRJ Brasil Hospital Pró-Cardíaco, Rio de Janeiro, RJ - Brasil; 10 Universidade Federal de São Paulo São PauloSP Brasil Universidade Federal de São Paulo, São Paulo, SP - Brasil; 11 Universidade Federal do Rio de Janeiro Hospital Universitário Clementino Fraga Filho Rio de JaneiroRJ Brasil Universidade Federal do Rio de Janeiro Hospital Universitário Clementino Fraga Filho, Rio de Janeiro, RJ - Brasil; 12 Centrocor VitóriaES Brasil Centrocor – Cardiologia, Vitória, ES - Brasil; 13 Hospital das Clinicas Universidade Federal de Pernambuco RecifePE Brasil Hospital das Clinicas da Universidade Federal de Pernambuco - Medicina Clínica, Recife, PE - Brasil; 14 Pronto Socorro Cardiológico de Pernambuco Universidade de Pernambuco RecifePE Brasil Pronto Socorro Cardiológico de Pernambuco (Procape) - Universidade de Pernambuco,Recife, PE - Brasil; 15 Hospital de Clínicas de Porto Alegre Porto AlegreRS Brasil Hospital de Clínicas de Porto Alegre, Porto Alegre, RS - Brasil; 16 Hospital Felício Rocho Belo HorizonteMG Brasil Hospital Felício Rocho,Belo Horizonte, MG - Brasil; 17 Universidade Federal de Juiz de Fora Juiz de ForaMG Brasil Universidade Federal de Juiz de Fora, Juiz de Fora, MG - Brasil; 18 Hospital Cardiológico Costantini Ltda CuritibaPR Brasil Hospital Cardiológico Costantini Ltda, Curitiba, PR - Brasil; 19 Hospital Marcelino Champagnat CuritibaPR Brasil Hospital Marcelino Champagnat,Curitiba, PR - Brasil; 20 Irmandade da Santa Casa de Misericórdia de Curitiba CuritibaPR Brasil Irmandade da Santa Casa de Misericórdia de Curitiba,Curitiba, PR - Brasil; 21 Universidade de São Paulo Faculdade de Medicina Hospital das Clínicas São PauloSP Brasil Universidade de São Paulo Faculdade de Medicina Hospital das Clínicas Instituto do Coração, São Paulo, SP - Brasil; 22 Universidade de São Paulo Hospital das Clínicas Faculdade de Medicina de Ribeirão Preto Ribeirão PretoSP Brasil Universidade de São Paulo Hospital das Clínicas da Faculdade de Medicina de Ribeirão Preto, Ribeirão Preto, SP - Brasil; 23 Complexo Hospitalar Santa Casa de Porto Alegre Porto AlegreRS Brasil Complexo Hospitalar Santa Casa de Porto Alegre, Porto Alegre, RS - Brasil; 24 Pontifícia Universidade Católica do Rio Grande do Sul Porto AlegreRS Brasil Pontifícia Universidade Católica do Rio Grande do Sul, Porto Alegre, RS - Brasil; 25 Universidade Estadual de Campinas Faculdade de Enfermagem CampinasSP Brasil Universidade Estadual de Campinas - Faculdade de Enfermagem, Campinas, SP – Brasil

**Keywords:** Dispositivos Cardíacos Eletrônicos Implantáveis/complicações, Ética Baseada em Princípios, Autópsia/métodos

## Abstract

O manejo de dispositivos cardíacos eletrônicos implantáveis de pacientes que evoluem a óbito tem sido motivo de controvérsia. Em nosso meio, não há recomendações uniformes, estando baseadas exclusivamente em protocolos institucionais e em costumes regionais. Quando o cadáver é submetido para cremação, além de outros cuidados, recomenda-se a retirada do dispositivo devido ao risco de explosão e dano do equipamento crematório. Principalmente no contexto da pandemia causada pelo SARS-Cov-2, a orientação e organização de unidades hospitalares e serviços funerários é imprescindível para minimizar o fluxo de pessoas em contato com fluidos corporais de indivíduos falecidos por COVID-19. Nesse sentido, a Sociedade Brasileira de Arritmias Cardíacas elaborou este documento com orientações práticas, tendo como base publicações internacionais e recomendação emitida pelo Conselho Federal de Medicina do Brasil.

O número de implantes de dispositivos cardíacos eletrônicos implantáveis (DCEI) (marcapassos, ressincronizadores cardíacos, cardioversores-desfibriladores implantáveis e monitores de eventos) tem aumentado consideravelmente nas últimas décadas devido ao grande avanço em seus recursos diagnósticos e terapêuticos, além do evidente aumento da longevidade dos pacientes (pacientes idosos podem ter maior longevidade graças aos DCEI). Dessa forma, o aumento de situações clínicas em que o uso desses dispositivos se aplica é acompanhado por aumento de complicações e outros problemas a eles relacionados.

Uma grande preocupação em relação aos DCEI, principalmente na população de idosos, em decorrência das taxas de mortalidade relacionadas à infecção pelo SARS-Cov-2 (COVID-19), é a conduta após o óbito:

- O DCEI deve ser desligado?
- O DCEI deve ser removido para o sepultamento?
- O cadáver vai ser cremado. Quais cuidados devemos tomar?
- No caso de remoção do DCEI, quem deve realizar o procedimento?

Respondendo a esses questionamentos, a Sociedade Brasileira de Arritmias Cardíacas (SOBRAC) elaborou algumas recomendações baseadas em evidências internacionais., que julgamos ser imprescindíveis para o esclarecimento de médicos, hospitais e serviços funerários que lidam diariamente com essa situação:

1. Em pacientes que faleceram de morte súbita, os DCEI devem ser submetidos a avaliação eletrônica quando o médico responsável pelo paciente considerar necessário o esclarecimento da causa da morte, uma vez que os dispositivos dispõem de recursos que possibilitam a gravação e o armazenamento do ritmo cardíaco nesses casos.
2. Em portadores de DCEI que serão sepultados, não se faz necessária a reprogramação ou remoção do aparelho.
3. Portadores de DCEI que serão submetidos à cremação devem, obrigatoriamente, ter seu aparelho removido devido ao risco de explosão causada pelo superaquecimento (cerca de 1.300°C por 90 minutos) e danos ao equipamento crematório.
4.  Os cardioversores-desfibriladores implantáveis (CDI) de cadáveres que serão cremados ou que serão submetidos a autópsia devem ter as terapias desabilitadas por telemetria, a fim de prevenir o disparo de choques durante a manipulação do CDI pelo profissional que realiza o trabalho. A reprogramação por telemetria deve ser realizada por médico com área de atuação em estimulação cardíaca eletrônica implantável ou por técnico devidamente qualificado. Para a autópsia, eventualmente se pode considerar, como alternativa em casos excepcionais, a colocação do imã sobre o gerador do CDI em vez da reprogramação por telemetria. Nesse caso, o imã deve ser mantido sobre o gerador mesmo após a sua retirada, uma vez que o risco de choque persiste até que as terapias sejam desabilitadas com programação eletrônica.
5. De acordo com ofício expedido pelo Conselho Federal de Medicina (anexo) em 05 de maio de 2020, atendendo à solicitação da SOBRAC, sob o número 2.628/2020–DEPCO, a retirada dos DCEI *post mortem* deve ser realizada “preferencialmente por médico e devidamente registrado no prontuário do paciente, resguardando a segurança no momento da pandemia COVID-19, por isso, a importância do envolvimento da diretoria técnica para criar o melhor protocolo para a instituição”. A SOBRAC alerta que o profissional não médico (seja do serviço funerário ou da própria unidade hospitalar) que eventualmente seja designado para essa tarefa deve ser devidamente treinado, ainda que tal procedimento exija técnica simples e não coloque em risco a vida do profissional que executa o procedimento.

O entendimento dos aspectos relacionados ao manuseio de DCEI após o óbito, em momento de pandemia pela COVID-19, é fundamental para reduzir o risco de disseminação da doença.

Abaixo, descrevemos a técnica de remoção do DCEI:

- paramentação apropriada para o procedimento, a fim de evitar a contaminação do profissional através de fluidos corporais;
- utilizar lâmina de bisturi para incisar a pele profundamente em cima do gerador de pulsos;
- divulsionar o tecido profundo com os dedos ou tesoura;
- o gerador deve ser puxado para fora, cortando-se os eletrodos com tesoura;
- fechar a incisão com gaze e esparadrapo, não sendo necessária sutura;
- descartar o dispositivo de forma segura, segundo recomendações técnicas e protocolos institucionais.
