# Supplementary material for: Recomendações para o Manejo de Dispositivos Cardíacos Eletrônicos Implantáveis Post Mortem
Source: Arq Bras Cardiol. 2020 Dec 1;115(6):1178–9. [Article in Portuguese] doi: 10.36660/abc.20200447 (PMC8133731; doi:10.36660/abc.20200447)
Supplement: Translation

# Translation: Recommendations for the Post-Mortem Management of Cardiac Implantable Electronic Devices

**Authors:** Júlio César de Oliveira, Alexsandro Alves Fagundes, Ricardo Alkmim-Teixeira, José Mário Baggio, Luciana Armaganijan, Andre d’Avila, Eduardo B. Saad, Veridiana Silva de Andrade, Luis Gustavo Belo de Moraes, Ricardo Kuniyoshi, André Gustavo da Silva Rezende, Mauricio Pimentel, Thiago da Rocha Rodrigues, Helio Lima de Brito, Elenir Nadalin, Cristiano Faria Pisani, Elerson Arfelli, Fatima Dumas Cintra, Carlos Antonio Abunader Kalil, Sissy Lara de Melo, Priscila Moreno Sperling Cannavan

**Affiliations:** 1 Brazil Universidade Federal de Mato Grosso, Cuiabá, MS - Brazil; 2 Brazil Hospital Geral Filantrópico Universitário de Cuiabá, Cuiabá, MS - Brazil; 3 Brazil Universidade do Estado da Bahia, Salvador, BA - Brazil; 4 Brazil Hospital Renascentista, Pouso Alegre, MG - Brazil; 5 Brazil Universidade do Vale do Sapucaí, Pouso Alegre, MG - Brazil; 6 Brazil Instituto de Cardiologia do Distrito Federal, Brasília, DF - Brazil; 7 Brazil Instituto Dante Pazzanese de Cardiologia, São Paulo, SP - Brazil; 8 Brazil Hospital SOS Cardio, Florianópolis, SC - Brazil; 9 Brazil Hospital Pró-Cardíaco, Rio de Janeiro, RJ - Brazil; 10 Brazil Universidade Federal de São Paulo, São Paulo, SP - Brazil; 11 Brazil Universidade Federal do Rio de Janeiro Hospital Universitário Clementino Fraga Filho, Rio de Janeiro, RJ - Brazil; 12 Brazil Centrocor – Cardiologia, Vitória, ES - Brazil; 13 Brazil Hospital das Clinicas da Universidade Federal de Pernambuco - Medicina Clínica, Recife, PE - Brazil; 14 Brazil Pronto Socorro Cardiológico de Pernambuco (Procape) - Universidade de Pernambuco, Recife, PE - Brazil; 15 Brazil Hospital de Clínicas de Porto Alegre, Porto Alegre, RS - Brazil; 16 Brazil Hospital Felício Rocho,Belo Horizonte, MG - Brazil; 17 Brazil Universidade Federal de Juiz de Fora, Juiz de Fora, MG - Brazil; 18 Brazil Hospital Cardiológico Costantini Ltda, Curitiba, PR - Brazil; 19 Brazil Hospital Marcelino Champagnat,Curitiba, PR - Brazil; 20 Brazil Irmandade da Santa Casa de Misericórdia de Curitiba, Curitiba, PR - Brazil; 21 Brazil Universidade de São Paulo Faculdade de Medicina Hospital das Clínicas Instituto do Coração, São Paulo, SP - Brazil; 22 Brazil Universidade de São Paulo Hospital das Clínicas da Faculdade de Medicina de Ribeirão Preto, Ribeirão Preto, SP - Brazil; 23 Brazil Complexo Hospitalar Santa Casa de Porto Alegre, Porto Alegre, RS - Brazil; 24 Brazil Pontifícia Universidade Católica do Rio Grande do Sul, Porto Alegre, RS - Brazil; 25 Brazil Universidade Estadual de Campinas - Faculdade de Enfermagem, Campinas, SP – Brazil

**Keywords:** Cardiac Resynchronization Therapy Device/complications, Principle Based Ethics, Autopsy/methods

## Abstract

The management of cardiac implantable electronic devices after death has become a source of controversy. There are no uniform recommendations for such management in Brazil; practices rely exclusively on institutional protocols and regional custom. When the cadaver is sent for cremation, it is recommended to remove the device due to the risk of explosion and damage to crematorium equipment, in addition to other precautions. Especially in the context of the SARS-CoV-2 pandemic, proper guidance and organization of hospital mortuary facilities and funeral services is essential to minimize the flow of people in contact with bodily fluids from individuals who have died with COVID-19. In this context, the Brazilian Society of Cardiac Arrhythmias has prepared this document with practical guidelines, based on international publications and a recommendation issued by the Brazilian Federal Medical Council.

The number of cardiac implantable electronic devices (CIEDs) placed, such as pacemakers, multisite devices, implantable cardioverter-defibrillators, and implantable loop recorder, has increased substantially in recent decades due to major diagnostic and therapeutic advances, as well as to the evident increases achieved in patient longevity (older adults may experience a longer life span as a result of CIED placement). The growing range of clinical situations in which these devices are used has been followed by an increase in complications and other related issues.

One major concern in relation to CIEDs—especially in the elderly population, due to the high mortality rates related to SARS-Cov-2 infection (COVID-19)—is their handling after death:

-  Should the CIED be turned off?
-  Should the CIED be removed before the patient’s burial?
-  If the body will be cremated, which precautions should be taken?
-  If the decision is made to remove the CIED, who should perform the procedure?

In response to these questions, the Brazilian Society of Cardiac Arrhythmias (SOBRAC) has developed a series of recommendations based on international evidence, which we believe to be essential guidance for physicians, hospitals, and funeral services, which are faced with this situation on a daily basis:

1. In cases of sudden death, the CIED should be electronically interrogated whenever the attending physician believes further elucidation of the cause of death is warranted, as the capability of these devices to record and store heart rhythm can be useful for this purpose.
2. In cadaver with CIEDs who will be buried rather than cremated, neither reprogramming nor device removal is necessary.
3. Conversely, cadaver with CIEDs whose remains will be cremated must always undergo device removal due to the risk of explosion and damage to crematorium facilities secondary to overheating (the device is exposed to a temperature of approximately 1300°C for 90 minutes).
4. Implantable cardioverter-defibrillators (ICDs) in cadaver who will be cremated or will undergo autopsy must be disabled by telemetry prior to the start of either of these procedures, so as to prevent inadvertent activation and delivery of a shock to forensic or mortuary professionals during manipulation. Device reprogramming via telemetry should be performed by a physician with expertise in cardiac implantable electronic devices or by a qualified technician. For autopsy, one alternative that can be considered under exceptional circumstances is magnet placement over the ICD generator instead of reprogramming via telemetry. In such cases, the magnet must be kept over the generator even after device removal, since the risk of shock persists until the ICD is disabled by reprogramming.
5. According to a guidance letter issued by the Federal Medical Council (see Appendix) on May 5, 2020, in response to a SOBRAC request, post-mortem removal of CIEDs should be carried out “preferably by a physician and duly noted in the patient’s medical record, as a safeguard during the time of the COVID-19 pandemic; hence, the importance of involving upper technical management so as to create the optimal protocol for each institution” (letter #2,628/2020 – DEPCO). SOBRAC warns that non-medical personnel (whether funeral directors or hospital staff) who may be assigned to this task must be properly trained to perform it, even though such a procedure is technically simple and does not endanger the life of the professional who performs it.

Understanding the aspects related to handling of CIEDs after death during the COVID-19 pandemic is essential to reducing the risk of disease spread.

The recommended CIED removal technique is as follows:

- don appropriate personal protective equipment in order to avoid contamination by bodily fluids;
- using a scalpel blade, deeply incise the skin overlying the pulse generator;
- divide the deep tissues bluntly (with fingers) or sharply (with scissors);
- pull out the generator and divide the electrodes with scissors;
- close the incision with gauze and tape—there is no need for sutures;
- dispose of the device safely, following the relevant technical recommendations and institutional protocols.
